# Biomechanical impact of enoxaparin sodium-chitosan-PMMA bone cement in hip arthroplasty: a preliminary finite element analysis

**DOI:** 10.3389/fsurg.2025.1615456

**Published:** 2025-06-24

**Authors:** Jianchao Chen, Xinzhe Ma, Zhiyong Li, Weiye Fan, Lijie Ma

**Affiliations:** Department of Orthopaedic Surgery, Hebei Medical University Third Hospital, Shijiazhuang, China

**Keywords:** finite element analysis, polymethyl methacrylate, bone cement, hip arthroplasty, biomechanical

## Abstract

**Objective:**

To determine the material parameters of PMMA and ES-CS-PMMA, and compare the stress distribution differences in hip arthroplasty postoperatively using finite element analysis.

**Methods:**

The Young's modulus and Poisson's ratio of PMMA and ES-CS-PMMA were calculated by measuring the propagation speed of ultrasound waves through the bone cements. Using CT images of a healthy adult male, a three-dimensional model of the natural femur and two postoperative 3D models (with PMMA and ES-CS-PMMA as adhesives) were established. Simulating slow walking motion, stress distribution differences in the cement sheath and femur were observed between the two postoperative models.

**Results:**

The Young's modulus of PMMA was 4,127 MPa with a Poisson's ratio of 0.25; ES-CS-PMMA exhibited an Young's modulus of 4,331 MPa and a Poisson's ratio of 0.28. In the two postoperative models, no significant differences were observed in stress distribution within the cement sheath or femur after prosthesis implantation. Compared to the natural femur, both PMMA and ES-CS-PMMA reduced cortical stress postoperatively, transferring stress to the femoral stem. In the distal region of the prosthesis, mean stress was significantly reduced post-implantation. In the midsection of the prosthesis, the implanted prosthesis bore higher peak stress than the natural femur.

**Conclusions:**

This study measured the material parameters of PMMA and ES-CS-PMMA. Compared to PMMA, using ES-CS-PMMA as an adhesive in hip arthroplasty did not alter the stress distribution of the cement sheath or femur post-implantation.

## 1 Introduction

Hip arthroplasty (HA) has been popularized worldwide since it was initiated in the 1960s, and the number of operations has increased rapidly (1). With the acceleration of population aging and the increase in the incidence of musculoskeletal diseases worldwide, hip arthroplasty has become an irreplaceable treatment for femoral neck fracture, femoral head necrosis, inflammatory arthritis and other diseases. Professor Charnley introduced Polymethyl methacrylate (PMMA) bone cement in the mid-20th century, and it has been widely used in the field of joint arthroplasty (2). PMMA bone cement has been pivotal to the early success of hip arthroplasty. However, failures of bone cement implants were historically misattributed to “bone cement disease”, a generalized critique that inadvertently promoted the rise of cementless prostheses. Advances in materials, design, and technology, have enhanced the durability of cementless fixation (3), leading to a global decline in cemented hip prosthesis usage. Nevertheless, evidence supporting superior clinical outcomes for cementless fixation remains inconclusive (4, 5). With increasing adoption of cementless implants, their limitations—particularly in elderly populations—have become apparent. Cementless fixation exhibits higher revision rates compared to cemented fixation in elderly patients (6–8), a trend corroborated by the 2018 Australian Orthopaedic Association National Joint Replacement Registry report, which identified significantly elevated revision risks for cementless prostheses in patients over 80 years old, especially within the first three postoperative months (9). Periprosthetic fractures, particularly involving femoral components, are the most common revision trigger. Burnett et al. reported that the surgical complication risk of cementless HA for femoral neck fracture is higher than cemented HA, with an 11-fold increased risk of periprosthetic fracture compared to cemented HA (10). These findings underscore PMMA's irreplaceable role in managing elderly patients with compromised bone quality or femoral neck fractures.

PMMA bone cement, however, poses systemic risks, including circulatory- and coagulation-system toxicity (11). Methyl methacrylate (MMA) monomer toxicity and exothermic polymerization-induced thermal necrosis synergistically damage bone cells and surrounding tissues. Necrotic debris, combined with histamine and prostaglandin release, exacerbates systemic dysregulation, elevating pulmonary embolism risks (12). Furthermore, thermal necrosis byproducts and cement particles provoke local inflammation, while excessive inflammatory responses and necrotic accumulation drive bone resorption at the bone-cement interface, increasing aseptic loosening risks.

To mitigate these adverse effects, Sun et al. developed enoxaparin sodium (ES)-loaded PMMA (ES-PMMA) by physically embedding ES into PMMA's porous matrix (13). ES-PMMA releases therapeutically relevant ES concentrations within 24 h, achieving antithrombotic efficacy via CD40 protein suppression in vascular endothelial cells (14). Additionally, ES-PMMA induces macrophage M2 polarization, enhances anti-inflammatory mediator secretion (15), and promotes interfacial osteogenesis, reducing loosening risks (16). Collectively, these studies validate ES's capacity to counteract PMMA's side effects.

Despite these advances, ES-PMMA suffers from burst-release kinetics and transient therapeutic windows. While effective for short-term thrombosis prevention, its rapid drug depletion fails to sustain the prolonged anti-inflammatory and osteogenic modulation required for bone repair. To address this, we incorporated chitosan (CS)—a cost-effective, biodegradable polymer with high drug-loading capacity (17)—as a sustained-release carrier. By conjugating ES with CS to form drug-loaded microspheres and integrating them into PMMA, we engineered enoxaparin sodium-chitosan-polymethyl methacrylate (ES-CS-PMMA) bone cement. Compared to ES-PMMA, ES-CS-PMMA exhibits attenuated burst release, prolonged release duration, and enhanced capacity to continuously suppress inflammation and stimulate peri-implant osteogenesis.

However, additive incorporation into bone cement alters its mechanical properties, potentially affecting *in vivo* stress distribution (18). Montserrat et al. mixed 3.5 g bupivacaine hydrochloride with 40.8 g PMMA and measured the mechanical properties of bone cement. The results showed that the addition of bupivacaine hydrochloride significantly reduced the flexural strength of bone cement, which fell short of the minimum flexural strength value (19). Kwong et al. added 6 g vancomycin to 43 g Copal PMMA cement and found that the flexural strength of the drug-loaded cement was significantly lower than the ISO standard (20). Finite element analysis (FEA), a tool superior to animal models for simulating physiological implant behavior, has been extensively applied in hip arthroplasty research. While prior FEA studies predominantly focus on prosthesis materials and designs (21, 22), biomechanical comparisons between PMMA and modified bone cements remain scarce, partly due to limited material parameter data. This study thus aims to characterize PMMA and ES-CS-PMMA material parameters and employ FEA to compare their stress distribution profiles post-hip arthroplasty, addressing a critical gap in functionalized bone cement optimization. As a preliminary study, this work establishes a computational framework that paves the way for future investigations, including multiscale modeling of long-term bone adaptation, integration of patient-specific dynamic loading patterns, and experimental validation through fatigue testing coupled with microstructural damage analysis. These extensions will enhance the clinical translatability of computational predictions in advanced bone cement development.

## 2 Materials and methods

### 2.1 Establishment of the FE model of hip arthroplasty

The femoral CT data of a healthy adult male were obtained from the professionally anonymized research case database of Hebei Medical University Third Hospital, in compliance with the Ethical Review Measures for Human Life Sciences and Medical Research [National Health Commission of China, No. 4 (2023), Article 32], which exempts studies using non-identifiable data from ethical approval. The generated cross-sectional CT images were stored in DICOM format and imported into Mimics 21.0 (Materialise, Leuven, Belgium) to extract femoral information and initially establish a rough three-dimensional model. Each part was exported to an STL-formatted data file. The reconstructed femoral STL file was subsequently imported into Geomagic 2021 (3D Systems, Rock Hill, USA) for advanced processing—including denoising, smoothing and accurate surface fitting—to generate a high-precision geometric solid model. The finalized model was exported in STP format.

The implant hip arthroplasty system (manufactured by Smith & Nephew PLC) utilized a titanium alloy construction comprising femoral shaft and femoral head components. A GOM Scan1 MV200 optical scanner (Zeiss Group, Oberkochen, Germany) performed comprehensive 3D surface mapping of the implant, with scan data archived in standardized STL format. Subsequent computational processing in UG 12.0 (Siemens PLM Software, Berlin & Munich, Germany) involved geometric refinement through surface denoising and mesh smoothing algorithms to generate a high-fidelity solid model, ultimately exported as STP-format files for biomechanical analysis.

**Figure 1:**
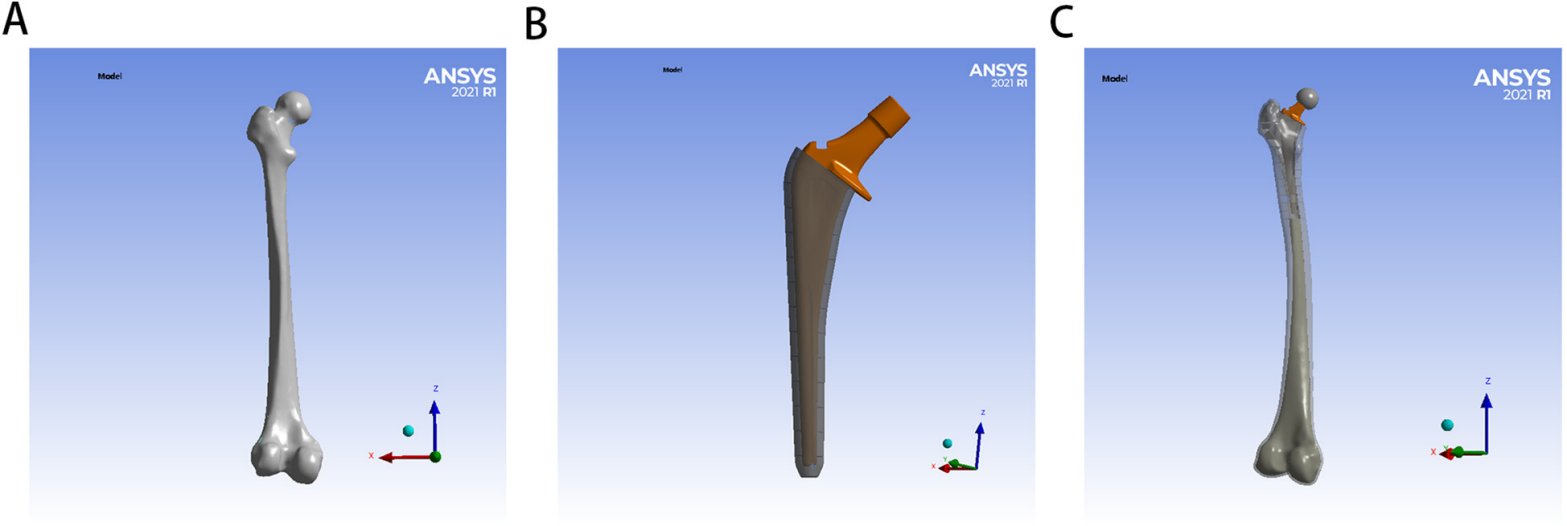
Establishment of FE model for hip arthroplasty: **(A)** for natural femur, **(B)** for implant with bone cement mantle, **(C)** for FE model for hip arthroplasty.

The surgical simulation models for hip arthroplasty were categorized into two experimental groups based on bone cement type: the PMMA group and the ES-CS-PMMA group. Femoral and implant STP-format models were imported into SolidWorks 2021 (Dassault Systèmes, Vélizy-Villacoublay, France) for assembly, with the femoral head being removed to facilitate stem insertion. The femoral stem was inserted into the proximal femur to keep the axis of the femoral stem and the femoral axis unified. The gap between the femoral stem and cortical bone was filled with different types of bone cement to form a cement mantle. The filling position was approximately 2 cm from the distal end of the femoral shaft, which was consistent with patients undergoing hip arthroplasty surgery. A pristine femoral model without stem insertion served as the anatomical control (Figure 1).

The geometric model of the hip arthroplasty construct was imported into ANSYS 21.0 (ANSYS Inc., Canonsburg, USA) for finite element mesh generation, establishing a computational model and generating post-force analysis data. Tetrahedral elements were implemented for mesh generation, with element density determined by anatomical feature complexity. A mesh sensitivity analysis was conducted, wherein critical regions were refined until stress convergence criteria (<5% variation in peak von Mises stress) were met. Excessive element counts escalate computational demands, while insufficient densities or suboptimal element quality compromise result accuracy. Through iterative refinement based on historical validation protocols, mesh parameters were optimized to enhance element quality and improve subsequent data reliability. Notably, the maximum relative difference in element counts between test groups was 4.5%, with identical meshing for PMMA and ES-CS-PMMA groups, ensuring cross-comparison validity. Quantitative nodal and elemental characteristics for each test group are systematically presented in Table 1.

**Table 1:** Numbers of nodes and elements for each model group.

Model	Node	Element
Control	1,093,104	722,554
PMMA	1,061,580	690,137
ES-CS-PMMA	1,061,580	690,137

### 2.2 Material parameters

FEA has become a useful tool for analyzing the structural stress of complex shapes, loads and material behaviors. It has been widely used in orthopedics, and the developed model has successfully predicted the mechanical properties of bone components. There are many precedents for the use of this method in the study of femoral head necrosis and hip arthroplasty (21, 22). However, previous studies have focused on the use of different prostheses and lack material parameters related to PMMA bone cement and the FEA of PMMA bone cement. Therefore, the first purpose of this study was to measure the Young's modulus and Poisson's ratio of different bone cements. The mechanical characteristics of PMMA bone cement and ES-CS-PMMA bone cement were evaluated through measurement of their Young's modulus and Poisson's ratio. Currently, no standardized testing methodology exists for determining these material properties in bone cement systems. Therefore, the ultrasonic testing methodology (GB/T 38897–2020) was referenced, which specifies two distinct approaches: the bulk wave method applicable to bulk solid specimens and the guided wave method designed for cylindrical filamentous samples. As bone cement constitutes a bulk solid material rather than filamentous structures, the bulk wave detection protocol was selected for this investigation.

The ultrasonic wave propagation velocity in solid materials is governed by their Young's modulus and density. On the basis of the known material density, the Young's modulus and Poisson's ratio of the material are calculated by measuring the propagation speed of ultrasonic waves in solid materials. First, the densities of the two kinds of bone cement were measured. The bone cement was made into a cubic sample of 1 cm^3^, and its mass was weighed with an electronic balance. Three samples were measured for each type of bone cement. The average density of the PMMA bone cement was 1,122 kg/m^3^, and that of the ES-CS-PMMA bone cement was 1,147 kg/m^3^.

The ultrasonic characterization was conducted using the UMS Advanced Ultrasonic Material Characterization System (Beijing, China) with system parameters configured as follows: 200 V excitation voltage, 5 MHz transducer frequency, and 20 dB signal gain. Longitudinal and shear wave propagation parameters were systematically evaluated through time-distance analysis, with wave velocities calculated from measured propagation times and path lengths. For each bone cement formulation, three independent specimens underwent ultrasonic testing with three random measurement locations per specimen to ensure statistical representation.

The longitudinal sound velocity of the tested sample was calculated according to following Equation (1). where *v*_*l*_ represents the sound velocity of the longitudinal wave (m/s), *n*_*l*_ represents the number of complete path propagations between the first echo signal and the last echo signal, h represents the thickness (m), and Δ*t*_*l*_ represents the time difference between the leading edge of the first echo signal and the leading edge of the last echo signal (s).

(1) $$V_{l}=\frac{2n_{l}h}{\Delta t_{l}}$$

The shear sound velocity of the tested sample was calculated according to following Equation (2). Where *v*_*s*_ represents the sound velocity of the shear wave (m/s), *n*_*s*_ represents the number of complete path propagations between the first echo signal and the last echo signal, h represents the thickness (m), and Δ*t*_*s*_ represents the time difference between the leading edge of the first echo signal and the leading edge of the last echo signal (s).

(2) $$V_{s}=\frac{2n_{s}h}{\Delta t_{s}}$$

The Young's modulus of the bone cement was obtained from the calculation of the longitudinal wave sound velocity, shear wave sound velocity and density.

The Young's modulus of the tested sample was calculated according to following Equation (3). where E represents the Young's modulus (Pa), *ρ* represents the density (kg/m^3^), *v*_*s*_ represents the sound velocity of the shear wave (m/s), and *v*_*l*_ represents the sound velocity of the longitudinal wave (m/s).

(3) $$E=\rho v_{s}^{2}\frac{3v_{l}^{2}-4v_{s}^{2}}{v_{l}^{2}-v_{s}^{2}}$$

The Poisson's ratio of the bone cement was obtained from the calculation of the longitudinal wave sound velocity and shear wave sound velocity.

The Poisson's ratio of the tested sample was calculated according to following Equation (4). where *μ* represents the Poisson's ratio, v_l_ represents the sound velocity of the longitudinal wave (m/s), and v_s_ represents the sound velocity of the shear wave (m/s).

(4) $$\mu =\frac{v_{l}^{2}-2v_{s}^{2}}{2(v_{l}^{2}-v_{s}^{2})}$$

According to the ultrasonic measurement results, the Young's modulus and Poisson's ratio of each sample were calculated, as shown in Table 2. In this study, according to the calculation results, the Young's modulus of the PMMA bone cement was 4,127 MPa, and the Poisson's ratio was 0.25. The Young's modulus of the ES-CS-PMMA bone cement was 4,331 MPa, and the Poisson's ratio was 0.28. The results show that adding chitosan drug-loaded microspheres to PMMA can increase the Young's modulus and Poisson's ratio. This provides a reference for this study and other scholars to study bone cement in the future. The mechanical properties of the other materials were obtained from previous studies (23–27). Table 3 details the parameters of each structural material used in the analysis, and all the material characteristics are simulated as a uniform linear elastic continuum with isotropic characteristics.

**Table 2:** Ultrasonic measurement results of different bone cements.

Sample	PMMA bone cement		ES-CS-PMMA bone cement	
	Young's modulus（GPa）	Poisson's ratio	Young's modulus（GPa）	Poisson's ratio
Sample 1	4.09033	0.245488	4.50145	0.266592
	4.14911	0.240381	4.45935	0.289598
	4.42371	0.272339	3.34896	0.296929
Sample 2	3.92258	0.244051	4.47295	0.27207
	4.00878	0.247252	4.35839	0.273002
	3.90540	0.246492	4.43806	0.28943
Sample 3	4.24955	0.232245	4.46886	0.273386
	4.21675	0.230449	4.32870	0.274530
	4.18007	0.250786	4.60039	0.271495
Mean ± SD	4.12736 ± 0.16607	0.24550 ± 0.01216	4.33075 ± 0.37646	0.27855 ± 0.01054

**Table 3:** Properties of the various components in the FE models.

Component name	Young's modulus（MPa）	Poisson's ratio
Femur cortical bone	15,000	0.30
Femur cancellous bone	1,100	0.30
Titanium alloy implant	110,000	0.30
PMMA bone cement	4,127	0.25
ES-CS-PMMA bone cement	4,331	0.28

### 2.3 Loading and boundary conditions

**Figure 2:**
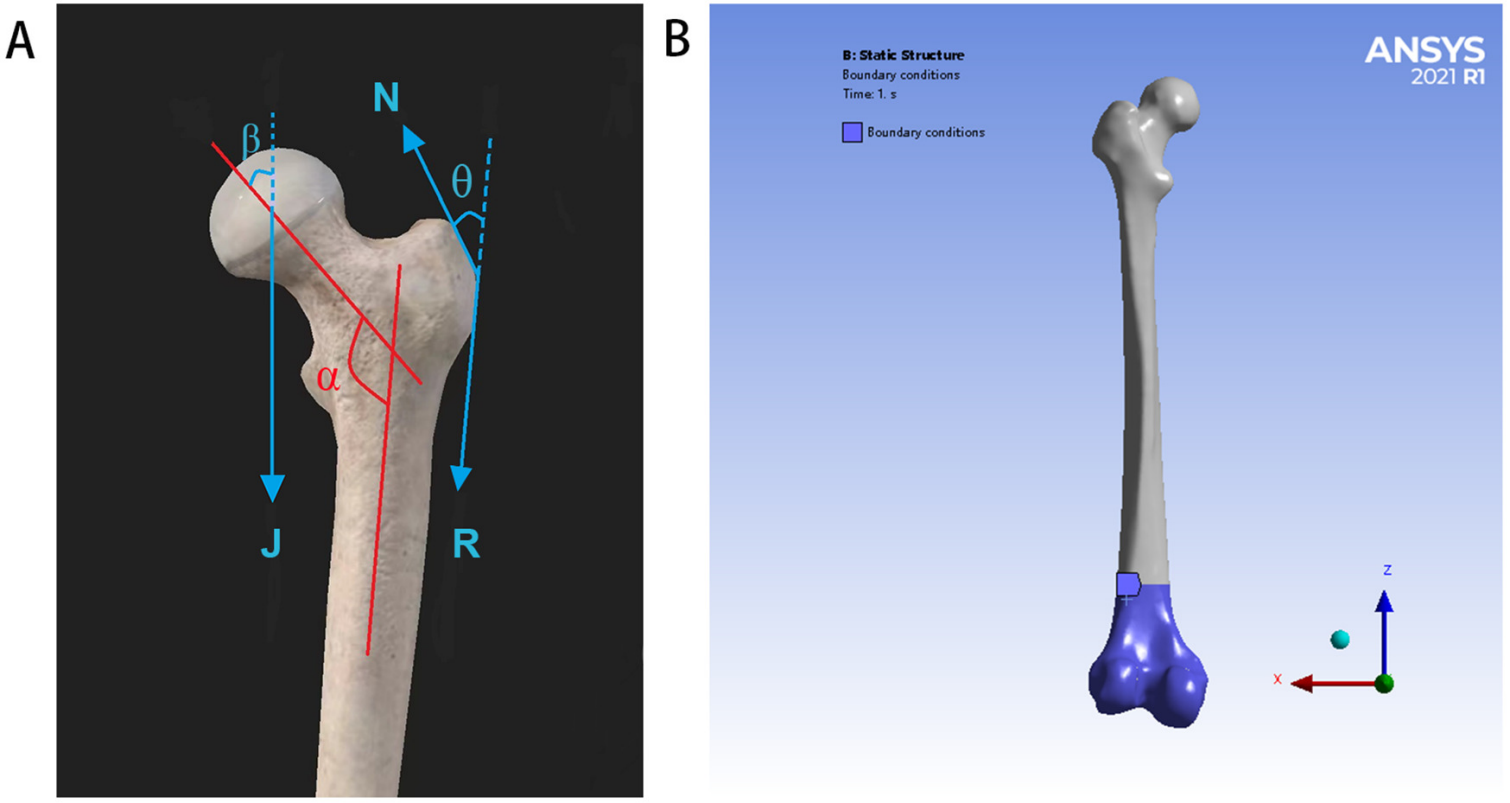
Load and boundary condition constraints: **(A)** for schematic diagram of simplified stress model of the proximal femur (J = 1,588 N, N = 1,039 N, R = 169 N, *α* = 135°, *β* = 29.5°, and *θ* = 24.4°), **(B)** for the distal femur is constrained in all directions, and the degree of freedom in all directions is zero.

In accordance with a previous report, the femoral model was simplified and assigned to simulate the slow walking force of an adult weighing 70 kg under the load of one foot (28). In the coronal plane, the neck shaft angle of the femur is 135°, the force of the femoral head (J) is 1,588 N, the loading position is the center of the femoral head, the direction is downward, and the included angle with the axis of the femoral neck is 29.5°. The pulling force (R) of the distal muscle group on the lateral trochanter is 169 N, which is downward and parallel to the axis of the femoral shaft. The pulling force (N) of the proximal muscle group on the lateral trochanter is 1,039 N, the direction is upward, and the included angle with the force R is 24.4°. According to the principles of the acting force and reaction force, the contact of all the models is a constraint relationship, the distal femur is constrained in all the directions, and the degree of freedom in all the directions is zero. Although these conditions simplify the model, they do not affect the validity of the results because the goal is to compare different models under consistent conditions. Therefore, the proposed results can be regarded as credible and objective. The load and boundary condition constraints are shown in Figure 2.

### 2.4 Stress analysis and measurement

Von Mises stress is a common scalar in the fields of mechanical engineering and materials science, and it is a standard for evaluating the performance of plates. This stress criterion can be used to evaluate the yield or failure of plates under complex load conditions.

The von Mises stresses in various parts of the model prosthesis and femur, including the total stress distribution nephogram, stress concentration area and peak stress of the prosthesis stem, femur and cement mantle, were recorded.

To analyze the stress distribution pattern of the FE model, the FE model was cut from the proximal end to the distal end in the Z-axis direction with a thickness of 10 mm until the prosthesis was completely divided. The mean value and peak value of the von Mises stress in each region are calculated. To further analyze the mean stress distribution around the femoral shaft, the femur was divided into proximal (regions 1–5), middle (regions 6–10) and distal (regions 11–15) regions.

### 2.5 Data analysis

The FEA results of the mean stress distribution and peak stress distribution are described and compared. To further analyze the mean stress distribution at the proximal, middle and distal regions of the three models, one-way ANOVA (with a Tukey's *post hoc* correction) was conducted. GraphPad Prism 10.0 (GraphPad Software, San Diego, USA) was used for data analysis and graphic representation. The significance level was set at 0.05.

## 3 Results

### 3.1 Von Mises stress distribution

**Figure 3:**
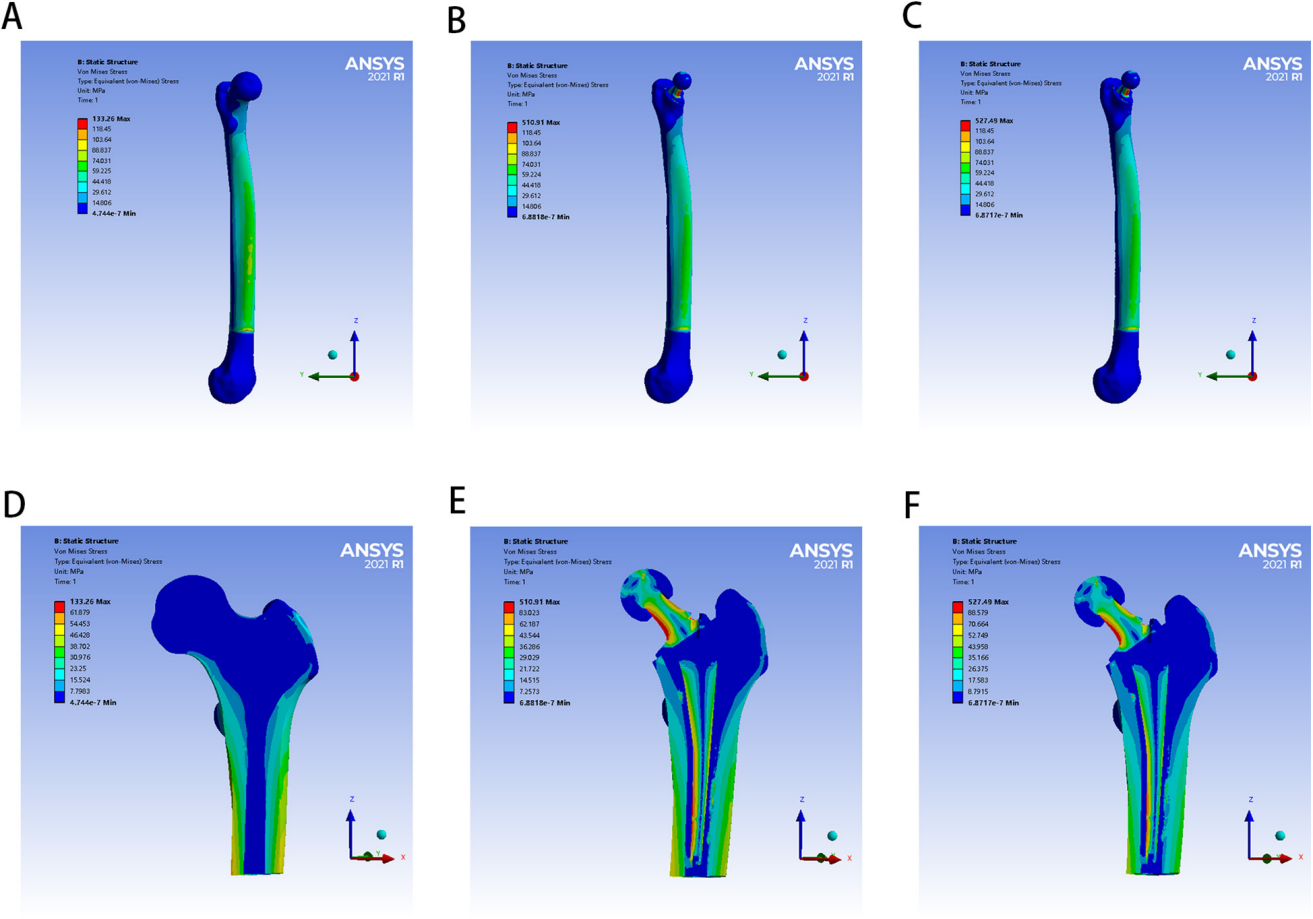
The von Mises stress distribution of femur in different FE models: **(A,D)** for control group; **(B,E)** for PMMA group; **(C,F)** for ES-CS-PMMA group.

**Figure 4:**
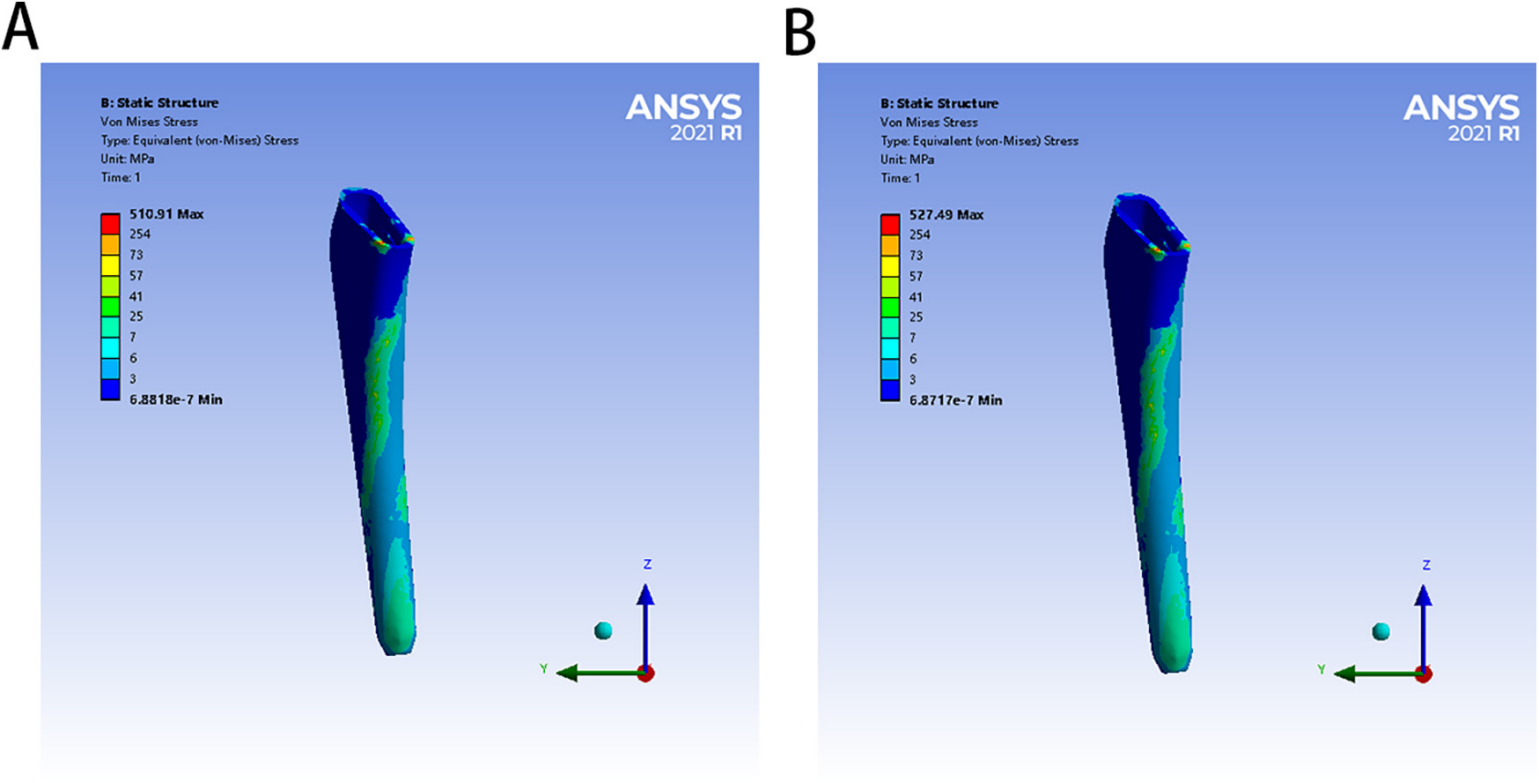
The von Mises stress distribution of two bone cement mantles in different FE models: **(A)** for PMMA group, **(B)** for ES-CS-PMMA.

**Figure 5:**
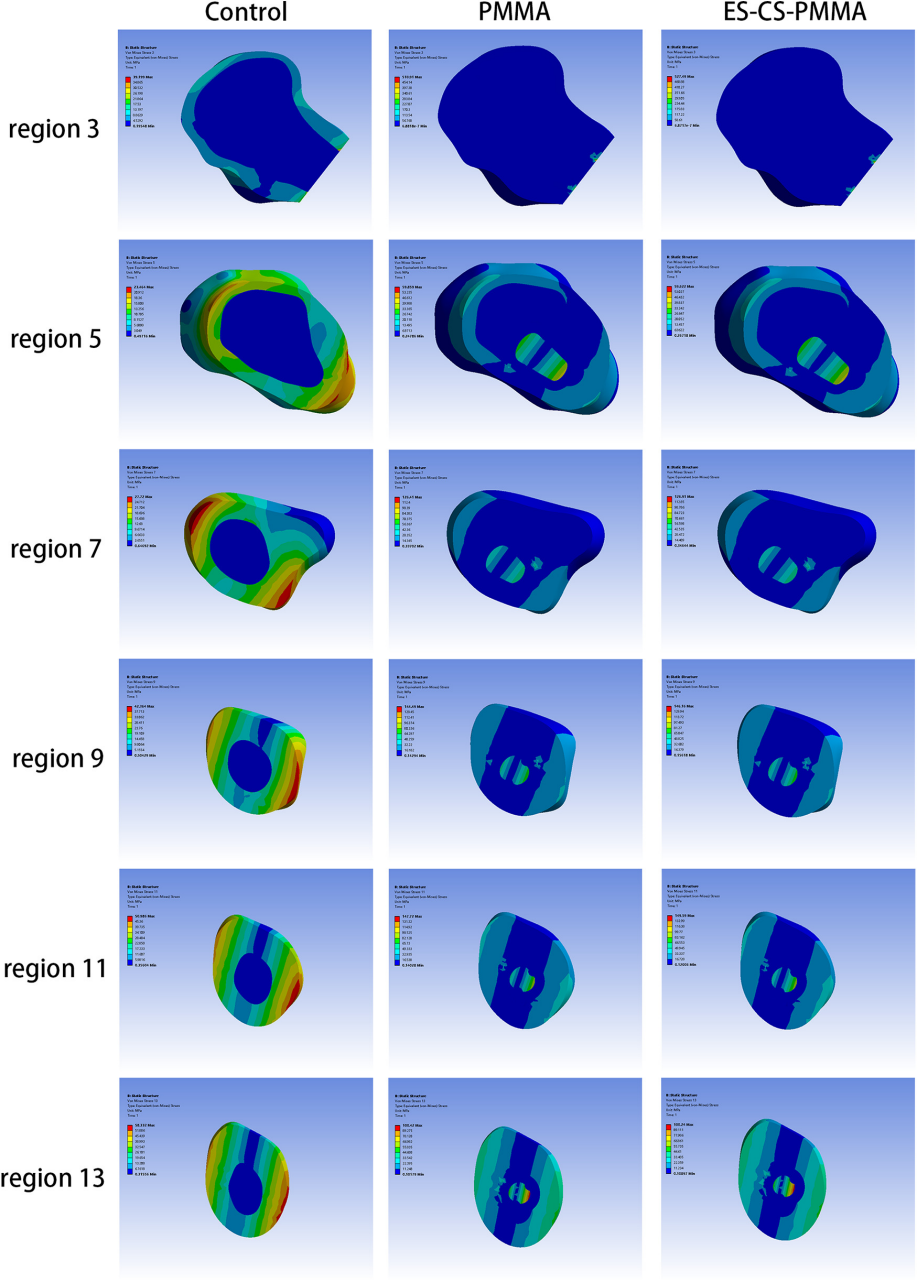
Transverse views of von Mises stress distribution in three FE models.

The von Mises stress distributions of the three models and two cement mantles are shown in Figures 3, 4. The results of the stress distribution for the different regions are given in Table 4 and depicted in transverse views in Figure 5.

**Table 4:** Mean and peak von Mises stresses.

Region	Mean stress (MPa)			Peak stress (MPa)		
	Control	PMMA	ES-CS-PMMA	Control	PMMA	ES-CS-PMMA
1	2.54	2.11	2.11	19.88	18.65	18.65
2	2.86	2.74	2.74	32.84	58.07	58.06
3	2.70	2.97	3.00	39.20	510.91	527.49
4	3.86	2.88	2.88	27.24	249.08	253.85
5	5.59	5.46	5.45	23.46	59.86	59.62
6	6.88	6.90	6.89	24.85	147.11	150.88
7	8.35	8.63	8.63	27.72	126.41	126.91
8	11.28	11.22	11.21	33.54	106.77	108.13
9	13.92	13.86	13.86	42.36	144.49	146.16
10	15.75	15.23	15.22	43.92	82.59	82.37
11	17.65	16.80	16.80	50.99	147.72	149.59
12	19.15	17.51	17.50	57.11	98.92	98.80
13	20.29	18.27	18.26	58.33	100.42	100.24
14	21.33	18.16	18.16	58.78	100.16	100.14
15	21.89	18.09	18.10	61.18	304.11	314.60

### 3.2 Mean von Mises stress

Considering the stress distribution of the two postoperative FE models, compared with that of the natural femur, the implantation of the prosthesis greatly changed the load transfer (Table 4 and Figures 3–5). In the natural femur, the stress is distributed mainly along the anteromedial and posterolateral sides and is concentrated mainly in the cortical bone (Figures 3, Figure 5). The cortical load gradually increased from the proximal region to the distal region (Table 4). After the prosthesis was implanted, the stress in the femur tended to decrease in both the PMMA group and the ES-CS-PMMA group, and the stress was transferred mainly through the femoral stem, such as in regions 5, 9 and 13 (Table 4 and Figure 5).

In the PMMA group and ES-CS-PMMA group, there was no significant difference in the stress distribution in the cement mantle between the two models (Figure 4).

**Figure 6:**
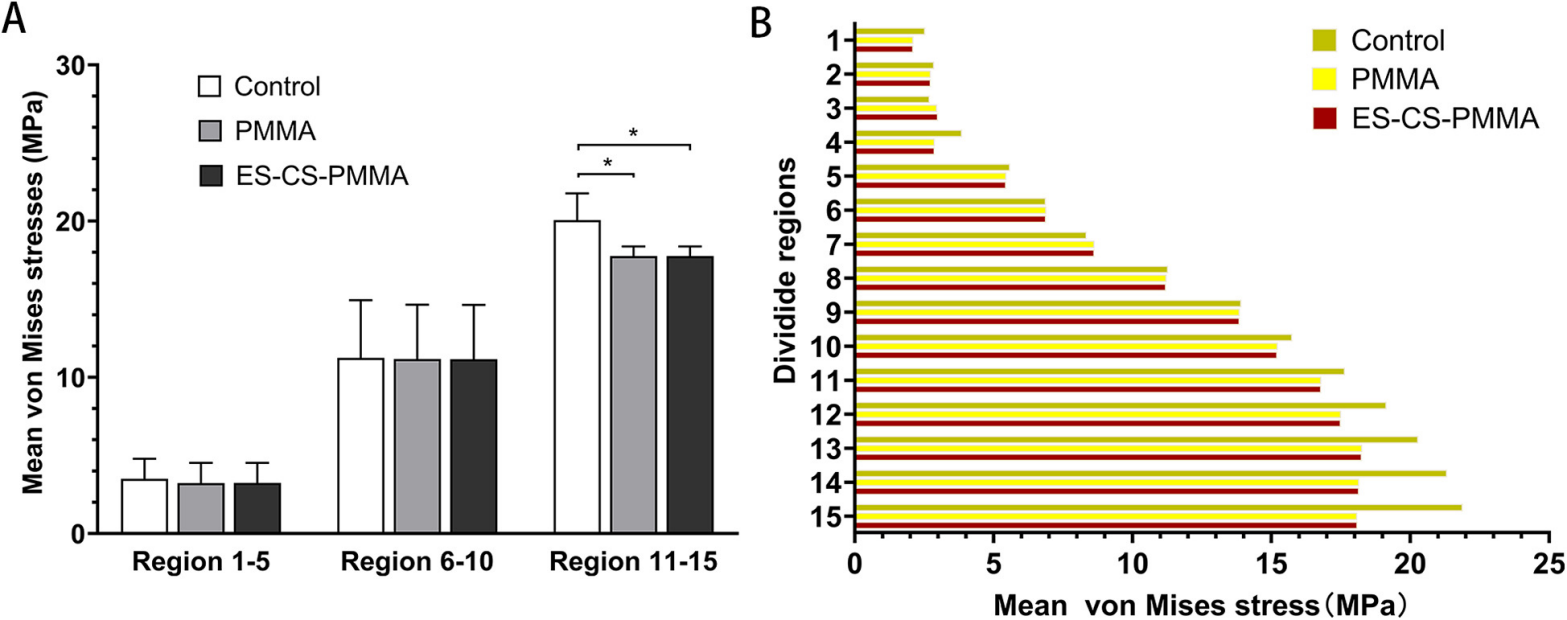
Mean von Mises stress and comparison of different femoral regions: **(A)** for mean stress distribution for different FE models in proximal (region 1-5), middle (region 6-10) and distal (region 11-15) femoral regions; **(B)** for mean stress distribution in three FE models (*p < 0.05).

As shown in Regions 1–10, although there is no significant difference in the mean stress between the proximal and middle regions of the three models (Table 5 and Figure 6), the stress in the proximal and middle regions of the prosthesis is unloaded after implantation, and the stress is transferred from the cortex to the prosthesis handle (Figure 5). In the distal region, as shown in Regions 11–15, the mean stress after prosthesis implantation decreased significantly (*p* < 0.05) (Table 5 and Figure 6).

**Table 5:** Mean von Mises stress and comparison of different femoral regions.

Group	Mean stress (MPa) (mean ± SD)	Comparison mean stress	*p*-value
Region 1–5			
Control	3.51 ± 1.27	Control vs. PMMA	0.9376
PMMA	3.23 ± 1.29	Control vs. ES-CS-PMMA	0.9393
ES-CS-PMMA	3.24 ± 1.28	PMMA vs. ES-CS-PMMA	>0.9999
Region 6–10			
Control	11.24 ± 3.70	Control vs. PMMA	0.9995
PMMA	11.17 ± 3.48	Control vs. ES-CS-PMMA	0.9994
ES-CS-PMMA	11.16 ± 3.48	PMMA vs. ES-CS-PMMA	>0.9999
Region 11–15			
Control	20.06 ± 1.71	Control vs. PMMA	0.0167
PMMA	17.77 ± 0.61	Control vs. ES-CS-PMMA	0.0166
ES-CS-PMMA	17.76 ± 0.62	PMMA vs. ES-CS-PMMA	>0.9999

### 3.3 Peak von Mises stress

**Figure 7:**
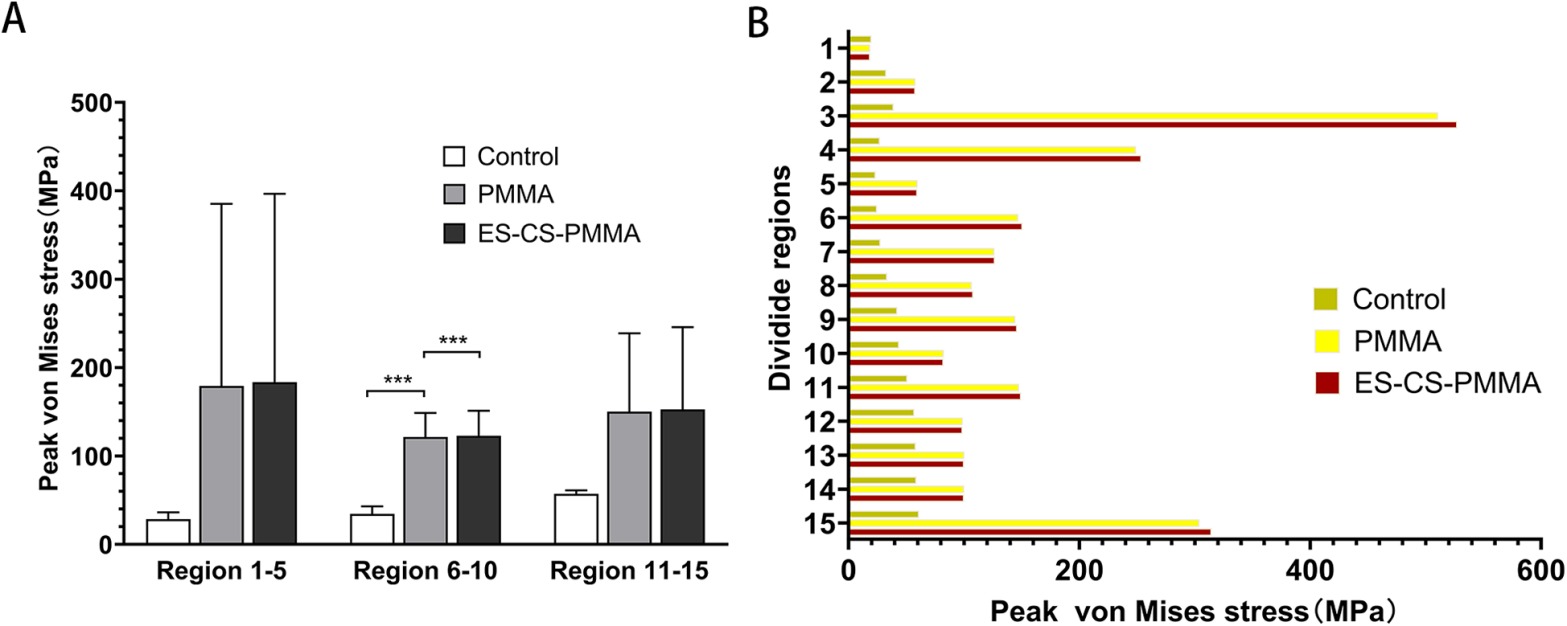
Peak von Mises stress and comparison of different femoral regions: **(A)** for peak stress distribution for different FE models in proximal (region 1-5), middle (region 6-10) and distal (region 11-15) femoral regions; **(B)** for peak stress distribution in three FE models (***p < 0.001).

After prosthesis implantation, the difference in load transfer is also reflected in the peak stress. For the natural femur, the peak stress appears on the anteromedial and posterolateral sides and gradually increases from the proximal region to the distal region, and the highest peak stress appears on the anteromedial side of the distal region (Table 4 and Figure 5). After implantation of the prosthesis, the highest peak stress occurred in proximal regions 3 and 4 and distal region 15 (Figure 7). In the middle region, the implant of the prosthesis resulted in greater peak stress than did the natural femur (*p* < 0.001) (Table 6 and Figure 7). However, there was no significant difference in peak stress between the two groups of models using different bone cements.

**Table 6:** Peak von Mises stress and comparison of different femoral regions.

Group	Peak stress (MPa) (mean ± SD)	Comparison mean stress	*p*-value
Region 1–5			
Control	28.52 ± 7.66	Control vs. PMMA	0.3748
PMMA	179.31 ± 205.92	Control vs. ES-CS-PMMA	0.3562
ES-CS-PMMA	183.53 ± 213.03	PMMA vs. ES-CS-PMMA	0.9992
Region 6–10			
Control	34.48 ± 8.52	Control vs. PMMA	0.0002
PMMA	121.47 ± 27.11	Control vs. ES-CS-PMMA	0.0002
ES-CS-PMMA	122.89 ± 28.29	PMMA vs. ES-CS-PMMA	0.9949
Region 11–15			
Control	57.28 ± 3.81	Control vs. PMMA	0.1590
PMMA	150.27 ± 88.47	Control vs. ES-CS-PMMA	0.1464
ES-CS-PMMA	152.67 ± 93.06	PMMA vs. ES-CS-PMMA	0.9985

## 4 Discussion

Hip arthroplasty has become essential for treating femoral neck fractures and joint diseases, with cemented prostheses regaining attention due to higher revision risks in cementless alternatives (29). While PMMA bone cement revolutionized joint arthroplasty, its exothermic reaction during implantation causes thermal necrosis, inflammation, and loosening (11, 12). Nevertheless, Modified bone cements, such as ES-PMMA, were developed to mitigate these effects by promoting osteogenesis and reducing inflammation (13–16). However, ES-PMMA's short release duration led to the creation of ES-CS-PMMA, which uses chitosan microspheres to prolong drug release and enhance biocompatibility. Despite these advantages, additives may compromise mechanical strength, necessitating thorough evaluation before clinical use to ensure surgical success.

The three-dimensional FE model shows that, compared with that of the natural femur, the stress distribution of the femur is different for both the PMMA bone cement and the ES-CS-PMMA bone cement. According to Wolff's law, a reduction in the bone load causes the bone to adapt itself by reducing its mechanical strength or decreasing its trabecular size (30). From a biomechanical point of view, the implanted prosthesis will inevitably redistribute the stress value of the femur. Subsequently, the reduced stress and debris induced around the prosthetic stem make the surrounding bones prone to osteoporosis and bone resorption. These two skeletal reactions usually worsen the stable environment of the prosthetic stem (31). However, in hip arthroplasty, prosthesis implantation inevitably leads to femoral load transfer. In the natural femur, the stress is mainly concentrated in the cortical bone, and the cortical load gradually increases from the proximal region to the distal region and is distributed along the anteromedial and posterolateral sides. When the prosthesis is implanted, the stress in the proximal and middle bone cortex is unloaded and transferred to the femoral shaft, but there is no significant difference in the mean stress among the three models. In the distal region, the mean stress of the femur after prosthesis implantation is significantly lower than that of the natural femur. The reduction in stress increases the risk of periprosthetic fractures in the distal region of the prosthesis to some extent, but this cannot be avoided in hip arthroplasty.

However, there was no significant difference in the femoral stress distribution between the two FE models, whether in the PMMA cement group or ES-CS-PMMA cement group. These findings indicate that adding an appropriate amount of CS microspheres or other substances to the bone cement does not significantly affect the stress distribution in the femur. In contrast, the addition of chitosan microspheres has a positive effect and reduces the side effects of bone cement during or after implantation. From a biomechanical point of view, these findings also indirectly confirm the efforts of other scholars in the modification of bone cement.

Nevertheless, comparison with other FEA studies must be interpreted cautiously due to the diversities in the loading conditions, bone-implant interface, age and weight of the subjects and experimental results.

Other FEA methods for hip arthroplasty focus mostly on simulating the static situation of standing on one leg after hip arthroplasty, and the applied force is relatively simple, which is different from the patient's postoperative motion state (32, 33). Aguilisa et al. applied a single force to the femoral head to compare the fatigue analysis results of different materials (26). This simplified loading condition ignores the influence of muscles and ligaments on the results. In patients after hip arthroplasty, walking slowly is more basic and commonly used than standing on one leg. In this study, we compared the effects of PMMA cement and ES-CS-PMMA cement on the femoral stress distribution after hip arthroplasty. Although applying a single force does not significantly reduce the accuracy, we still consider the role of the iliopsoas muscle and abductor muscle in exercise in the FE models to minimize the impact of load conditions on the research results. Although our model incorporates to simulate slow walking—surpassing single-leg stance simplifications—it does not fully replicate the multidirectional loading environment of the hip. Critical omissions include twisting force and dynamic shear forces. These simplifications likely underestimate shear stress at the stem-cement interface and obscure direction-specific micromotion patterns, particularly affecting stability assessments of anti-rotation implant designs. Nevertheless, since the core objective was comparing biomechanical responses between PMMA and ES-CS-PMMA cement under identical loading simplifications, such systematic bias minimally impacts the intergroup difference conclusions.

Regarding interfacial modeling, frictional face-to-face contact and frictionless node-to-node contact are used to describe the bone-implant interface. In this study, the implant–bone cement and bone cement–bone interfaces are completely bonded, which does not consider the micromotion of the implant. This idealized assumption may lead to an over-simplified characterization of interfacial stress transfer mechanisms. Interfacial micromotion can induce dynamic stress redistribution: at the proximal bone cement–bone interface, the fully bonded assumption may result in an underestimation of shear stress; conversely, at the distal stem–bone cement interface, this assumption could overestimate the peak compressive stress (34). Consequently, our findings are more applicable to evaluating the prosthesis mechanical performance during initial fixation, but cannot accurately reflect long-term effects such as micromotion-induced bone remodeling or bone cement fatigue failure risks. Subsequent research should integrate multimodal loading conditions and employ frictional contact models to quantify interface slippage effects on mechanical responses.

Although the FE analysis adopted gait loading for a 70 kg adult male, patient weight and age critically alter the biomechanical response. Increasing weight elevates dynamic hip joint force substantially, raising cement mantle stress significantly with interface micromotion potentially exceeding the bone resorption threshold (35). Elderly patients exhibit markedly lower trabecular bone modulus, increasing proximal femoral stress shielding and intensifying shear stress concentration at stem-cement interfaces (36). These findings indicate that obese patients require high-strength cement with optimized fixation. Although our fixed-parameter model reveals cement performance trends, future weight-age gradient models should quantify failure probability in high-risk cohorts.

Importantly, the FE predictions provide targeted guidance for subsequent experiments: Comparable cement mantle stress distributions between ES-CS-PMMA and PMMA groups suggest similar fatigue failure mechanisms in the modified cement. This consistency indicates that *in vitro* testing should prioritize monitoring interface crack propagation beyond 1 million cycles over short-term strength degradation. Limitations regarding omitted dynamic loads will be addressed in validation studies using hexapod simulators to replicate multi-axis loading environments, establishing a comprehensive biomechanical evaluation framework.

## 5 Conclusions

In summary, the Young's modulus and Poisson's ratio of PMMA bone cement and ES-CS-PMMA bone cement were measured in this study, laying a foundation for follow-up studies of bone cement implants or bone cement hip arthroplasty. The Young's modulus of PMMA was 4,127 MPa with a Poisson's ratio of 0.25; ES-CS-PMMA exhibited an Young's modulus of 4,331 MPa and a Poisson's ratio of 0.28. Moreover, regardless of whether PMMA bone cement or ES-CS-PMMA bone cement is used as an adhesive for hip arthroplasty, the stress distribution of the cement sheath and femur after prosthesis implantation has not changed. This finding shows that adding a proper amount of drugs or other active substances into bone cement does not reduce the mechanical properties of the bone cement.

## Data Availability

The original contributions presented in the study are included in the article/Supplementary Material, further inquiries can be directed to the corresponding author.
